# Cytoscape.js 2023 update: a graph theory library for visualization and analysis

**DOI:** 10.1093/bioinformatics/btad031

**Published:** 2023-01-16

**Authors:** Max Franz, Christian T Lopes, Dylan Fong, Mike Kucera, Manfred Cheung, Metin Can Siper, Gerardo Huck, Yue Dong, Onur Sumer, Gary D Bader

**Affiliations:** The Donnelly Centre, University of Toronto, Toronto, ON, Canada; The Donnelly Centre, University of Toronto, Toronto, ON, Canada; The Donnelly Centre, University of Toronto, Toronto, ON, Canada; The Donnelly Centre, University of Toronto, Toronto, ON, Canada; The Donnelly Centre, University of Toronto, Toronto, ON, Canada; Department of Molecular and Medical Genetics, School of Medicine, Oregon Health & Science University, Portland, OR, USA; The Donnelly Centre, University of Toronto, Toronto, ON, Canada; The Donnelly Centre, University of Toronto, Toronto, ON, Canada; Memorial Sloan Kettering Cancer Center, New York, NY, USA; The Donnelly Centre, University of Toronto, Toronto, ON, Canada; Department of Molecular Genetics, University of Toronto, Toronto, ON, Canada; Department of Computer Science, University of Toronto, Toronto, ON, Canada; The Lunenfeld-Tanenbaum Research Institute, Sinai Health System, Toronto, ON, Canada; Princess Margaret Cancer Centre, University Health Network, Toronto, ON, Canada

## Abstract

**Summary:**

Cytoscape.js is an open-source JavaScript-based graph library. Its most common use case is as a visualization software component, so it can be used to render interactive graphs in a web browser. It also can be used in a headless manner, useful for graph operations on a server, such as Node.js. This update describes new features and enhancements introduced over many new versions from 2015 to 2022.

**Availability and implementation:**

Cytoscape.js is implemented in JavaScript. Documentation, downloads and source code are available at http://js.cytoscape.org.

**Supplementary information:**

Supplementary data are available at *Bioinformatics* online.

## 1 Introduction

Cytoscape.js is a technology that can be embedded within web-apps, websites and servers that enable users to interact with networks for visualization and analysis. It is a critical software component used in research fields such as biology, sociology, computer security, cloud computing and data science. Cytoscape.js is used in research both as a tool, e.g. to create exploratory interactive visualizations in web applications (apps), and as a data representation, e.g. used to store protein–protein interaction network data. On GitHub, the most widely used platform for distributing open-source projects, Cytoscape.js is in the top 0.01% of software packages by popularity measured by number of user stars. Cytoscape.js is used by research groups (e.g. Ensembl, FlyBase and WormBase) (Drysdale, 2008; Harris *et al.*, 2010; Howe *et al.*, 2021), governmental organizations [e.g. the National Oceanic and Atmospheric Administration (USA), the National Security Agency (USA) and the National Health Service (UK)] and commercial organizations [e.g. Google, QuantStack (Jupyter) and Plotly] (https://js.cytoscape.org/#introduction/who-uses-cytoscape.js and https://github.com/cytoscape/cytoscape.js/network/dependents). Here, we provide an overview of the improvements and additions to Cytoscape.js since its original publication (Franz *et al.*, 2016), including details about releases, user adoption, major updates in version 3, performance, usability improvements, extensions and example use cases.

## 2 Implementation updates

### 2.1 Releases and user adoption

Overall, Cytoscape.js has had 226 software versions published, with 177 of them made after the initial publication (Franz *et al.*, 2016). We currently use a weekly cadence for patch releases and a monthly cadence for feature releases. Each week, a patch release is made if there are any bug fixes that have been made in the previous week. Similarly, a feature release is published if new features have been added during that month. This release schedule gives users a quick turnaround time for feedback (e.g. bug reports), and it provides a predictable and timely framework that the community can use when making third-party contributions. Cytoscape.js has consistently accelerated its adoption rate, with exponential growth of year-over-year user installations, since its initial publication in 2015 (https://npm-stat.com/charts.html?package=cytoscape&from=2011-01-30&to=2022-11-30).

### 2.2 Version 3

The initial publication of Cytoscape.js described version 2 of the software. Since then, we have released version 3, which breaks with some of the conventions of version 2. These incompatibilities were made in version 3 in order to provide integrations with current browser technology standards, such as the ECMAScript (ES) 2015+ specifications. Breaking changes were also made in version 3 to enable improvements that would help users to avoid making common coding errors. Additionally, we made changes to the API based on our experience with version 2 and based on feedback that we received from version 2 users. To minimize the effort required for upgrading from version 2 to version 3, we published an upgrade guide with a summarized list of all of the changes in version 3 as well as explicit instructions and examples for accommodating each change (https://blog.js.cytoscape.org/2017/04/11/3.0.0-release).

### 2.3 Usability

Usability is one of the most important priorities of Cytoscape.js, focusing both on app end-users and app authors. Since the initial publication, improvements have been made to the usability of gestures (e.g. easier, precise node and edge selection in dense networks). We have also added several new network layouts, such as ELK and Dagre (https://github.com/cytoscape/cytoscape.js-elk and https://github.com/cytoscape/cytoscape.js-dagre). Support for standard web frameworks, such as React (https://reactjs.org), eases the burden of integration of Cytoscape.js into new projects and integrations into standard web technologies (e.g. iterators) make it easier to integrate Cytoscape.js with modern JavaScript. We have improved our developer documentation based on feedback, including developer tool support (e.g. ES module support and console warning messages to mitigate issues early). Finally, we have made many improvements to the styling that Cytoscape.js supports, with more than double the number of supported style properties as compared to the initial publication—enabling users to be more expressive with their networks.

### 2.4 Extensions

Extensions are a key component of Cytoscape.js. They enable anyone to make additions to the application programming interface (API) of Cytoscape.js (e.g. layouts), and they can also be used to add reusable user interface elements to Cytoscape.js network visualizations. The number of extensions has grown to 67 (from 15 for our initial publication), many of which are popular network layout algorithms.

Anyone is free to make a Cytoscape.js extension, and these can immediately be used in projects, as there is no approval process required. Although extensions may be created privately, we encourage all organizations to publish their extensions as open-source code and to notify us of publication. When we are notified that a new extension has been published, we list it in our documentation to make it easier for users to find it.

## 3 Use cases

### 3.1 Systems Biology Graphical Notation

**Fig. 1.**
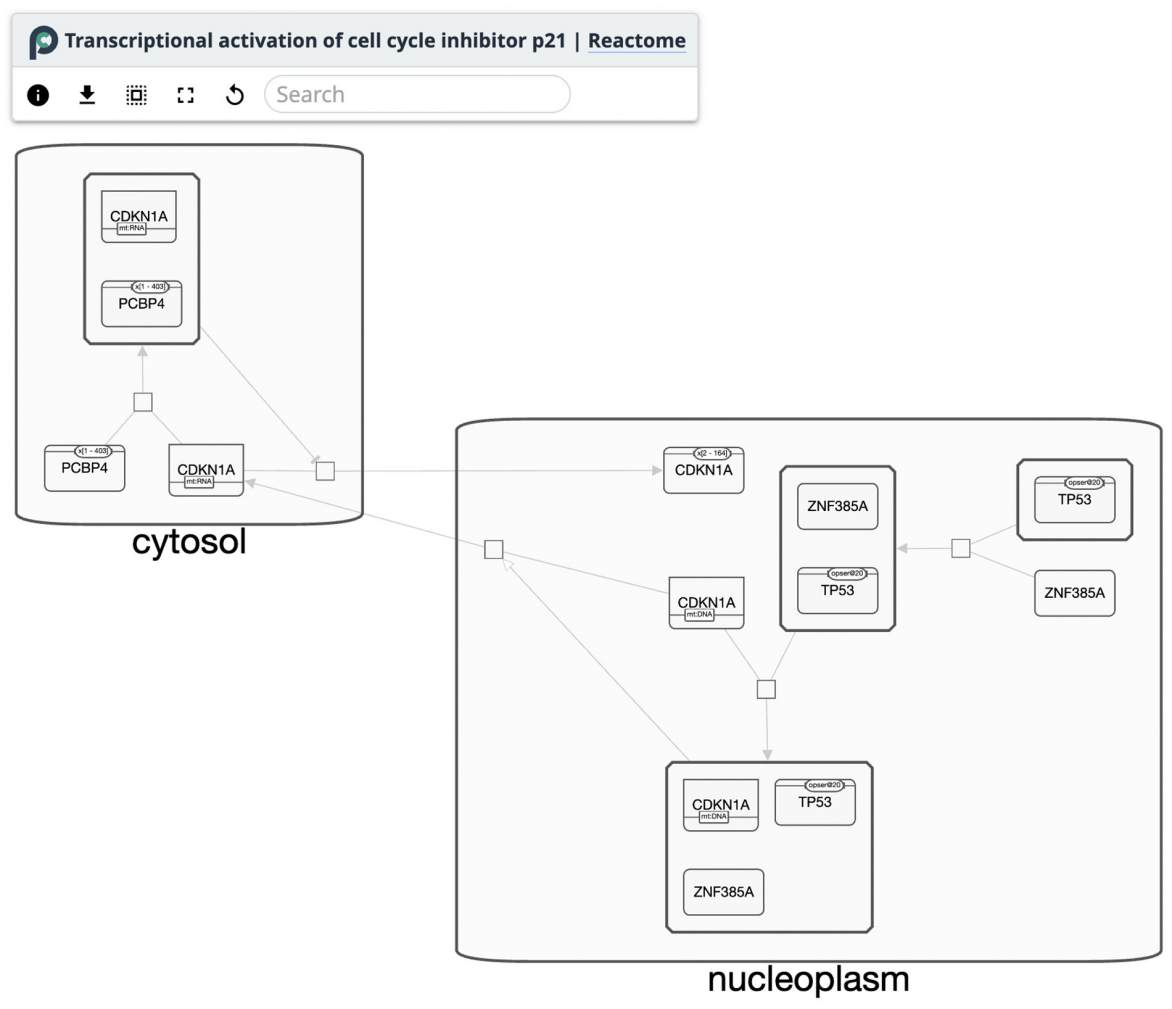
SBGN as used in Pathway Commons. This demonstrates the use of Cytoscape.js for interactive SBGN visualization of the ‘Transcriptional activation of cell cycle inhibitor p21’ pathway from Reactome, via a BioPAX to SBGNML conversion and a use of a Cytoscape.js SBGN stylesheet extension (Bergmann *et al.*, 2020; Cerami *et al.*, 2011; Demir *et al.*, 2013; Gillespie *et al.*, 2022; Le Novère *et al.*, 2009) (https://github.com/PathwayCommons/cytoscape-sbgn-stylesheet)

Cytoscape.js supports the visualization of Systems Biology Graphical Notation (SBGN) networks, and the interactive features of Cytoscape.js can be used to make SBGN editors and explorers. For example, the NEWT SBGN editor (Balci *et al.*, 2021) enables the user to create sophisticated SBGN diagrams interactively rather than programmatically. Another example is Pathway Commons, which supports the exploration of published SBGN networks via search and network exploration (Fig. 1).

### 3.2 Simple interaction drawing apps

By combining the interactive features of Cytoscape.js with editing extensions (e.g. ‘edgehandles’, ‘compound-drag-and-drop’), rich, easy-to-use network drawing tools can be created. Biofactoid (Wong *et al.*, 2021) is one such editor that empowers wet lab researchers to draw the biological interactions in their paper so that the corresponding interaction network data for their paper can be recorded and disseminated without any need for curation or computational training.

### 3.3 Data science

Cytoscape.js is connected to several notebook technologies to provide support for data science. Networks can be visualized using Cytoscape.js in R notebooks, Jupyter notebooks and Plotly dashboards. For instance, a data scientist can perform analysis using NetworkX in a Jupyter notebook, get the Cytoscape network JSON (JavaScript Object Notation) file from NetworkX and visualize the Cytoscape.js network in an output block in the notebook (https://github.com/cytoscape/ipycytoscape).

Figures that demonstrate the use of Cytoscape.js for the above use cases are included in Supplementary Figures S1–S3.

## 4 Future direction

We plan to continually improve Cytoscape.js based on feedback from the community. Further, additional features and enhancements will be made to accommodate an increasingly important research use cases such as live collaboration in the manner of Google Docs, the democratization of computational methods to non-computational researchers via easy-to-use apps, and the development of research-enabling and research-accelerating apps.

## Supplementary Material

btad031_Supplementary_Data

## Data Availability

Cytoscape.js source code and documentation are available at https://github.com/cytoscape/cytoscape.js.
