# Supplementary material for: Cytoscape.js 2023 update: a graph theory library for visualization and analysis
Source: Bioinformatics. 2023 Jan 16;39(1):btad031. doi: 10.1093/bioinformatics/btad031 (PMC9889963; doi:10.1093/bioinformatics/btad031)
Supplement: btad031_Supplementary_Data [file btad031_supplementary_data.zip › Cytoscape 2023 update - supplementary matterials/Supplementary materials.docx]

**Cytoscape.js 2023 update: supplementary materials**


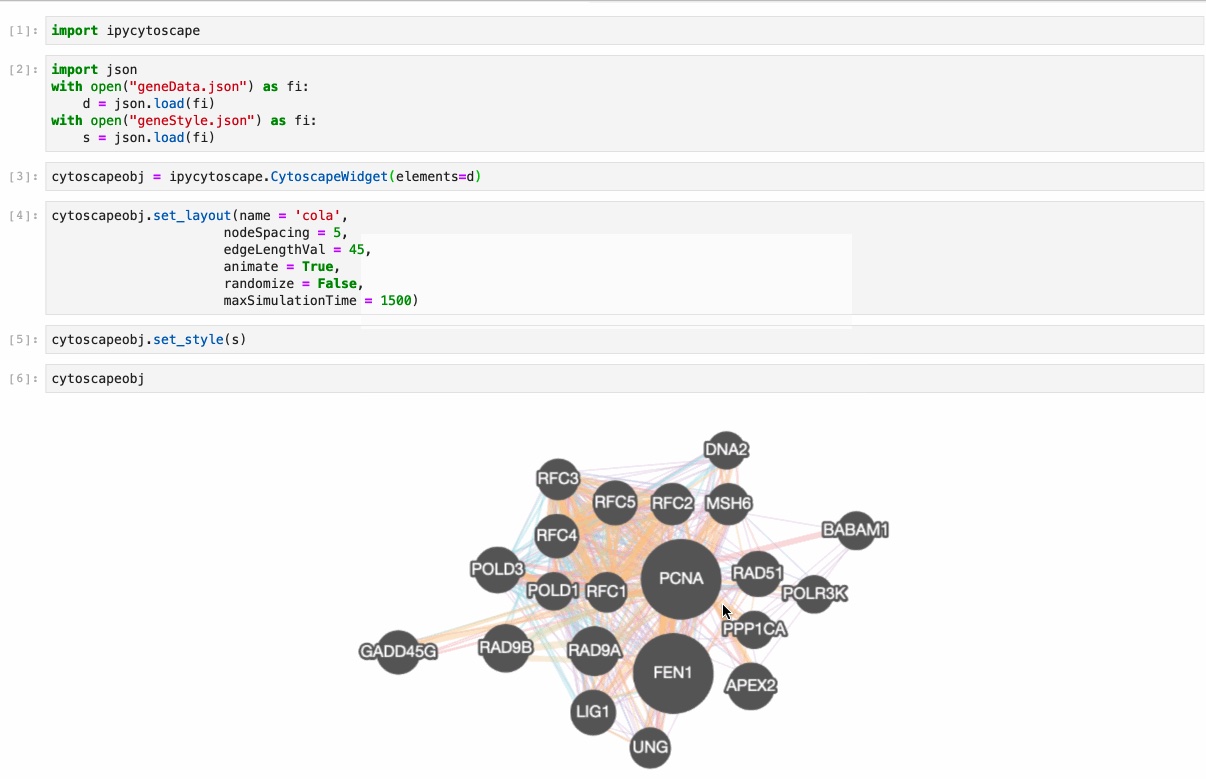


**Fig. S1. Cytoscape.js networks visualised in ipycytoscape.** This demonstrates the use of Cytoscape.js for interactive network visualisation in output blocks of Jupyter, using ipycytoscape (<https://github.com/cytoscape/ipycytoscape>).


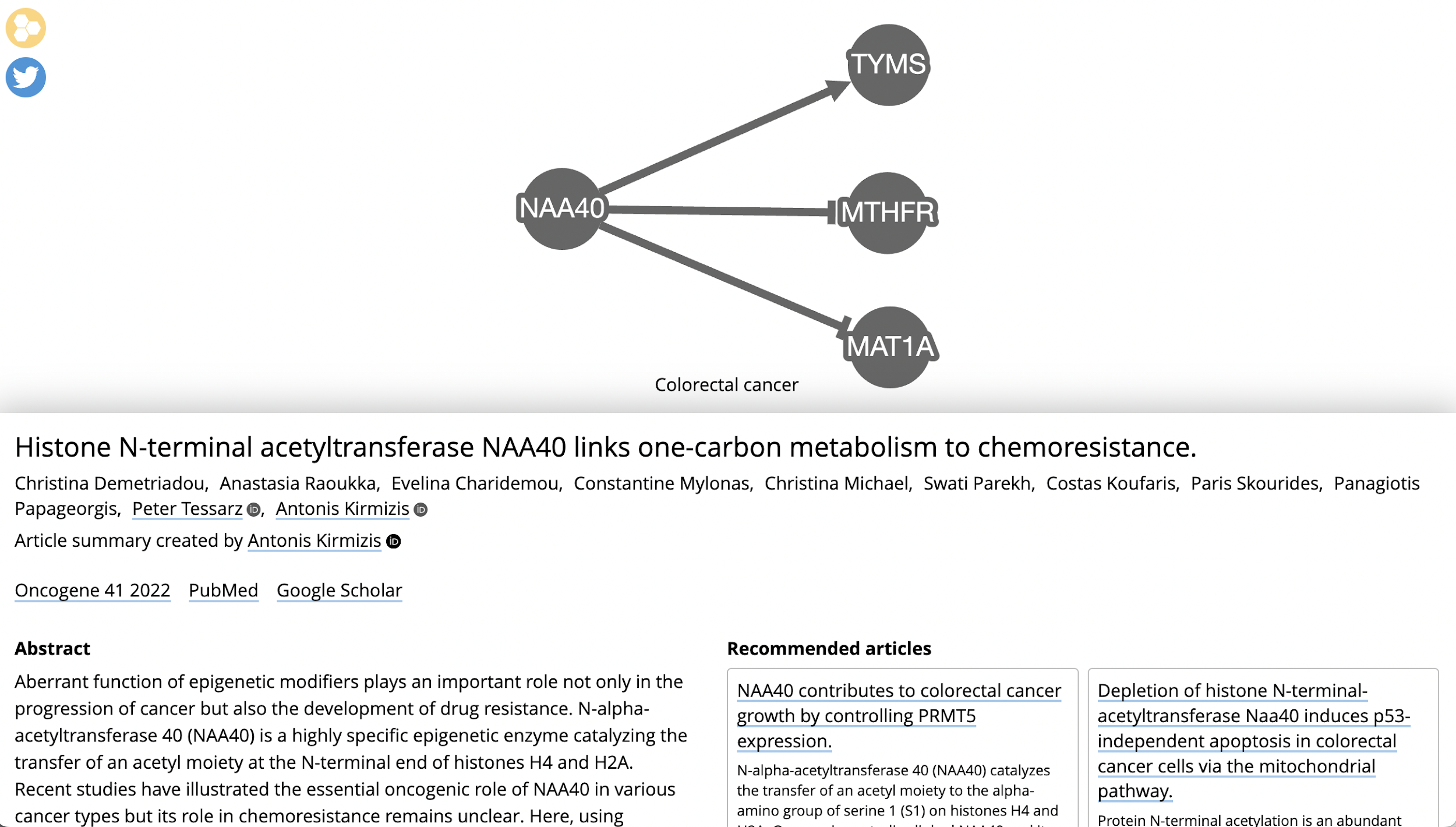


**Fig. S2. Biological interaction drawing in Biofactoid.** This demonstrates the use of Cytoscape.js for interactively drawing biological interactions. Researchers draw the interactions in their papers in order to create data representations of the findings in their papers, which can be exported into BioPAX.


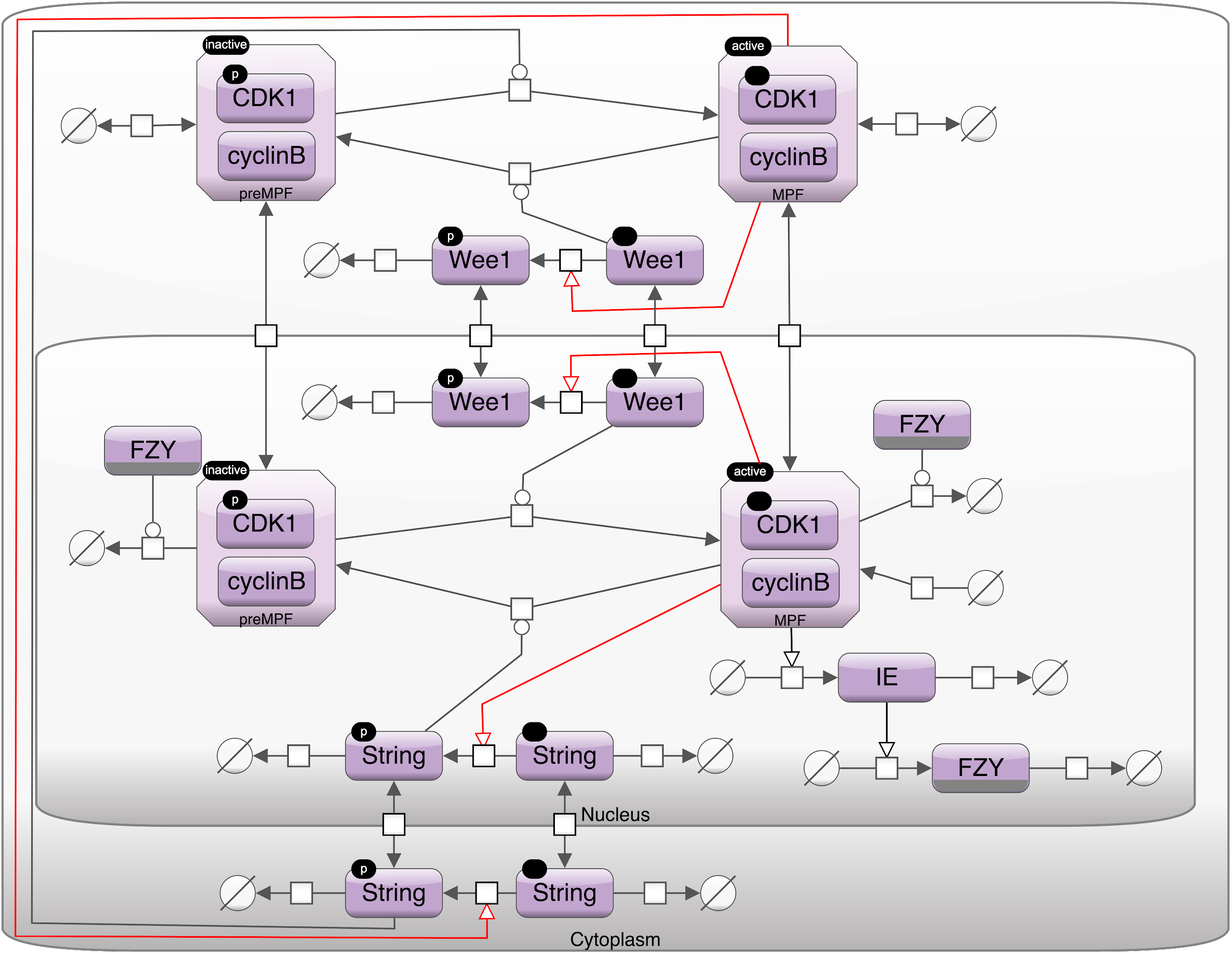
**Fig. S3. SBGN as used in NEWT.** This demonstrates the use of Cytoscape.js for interactive SBGN editing in the NEWT app.
