# Supplementary figures and images for: Cytoscape.js 2023 update: a graph theory library for visualization and analysis
Source: Bioinformatics. 2023 Jan 16;39(1):btad031. doi: 10.1093/bioinformatics/btad031 (PMC9889963; doi:10.1093/bioinformatics/btad031)

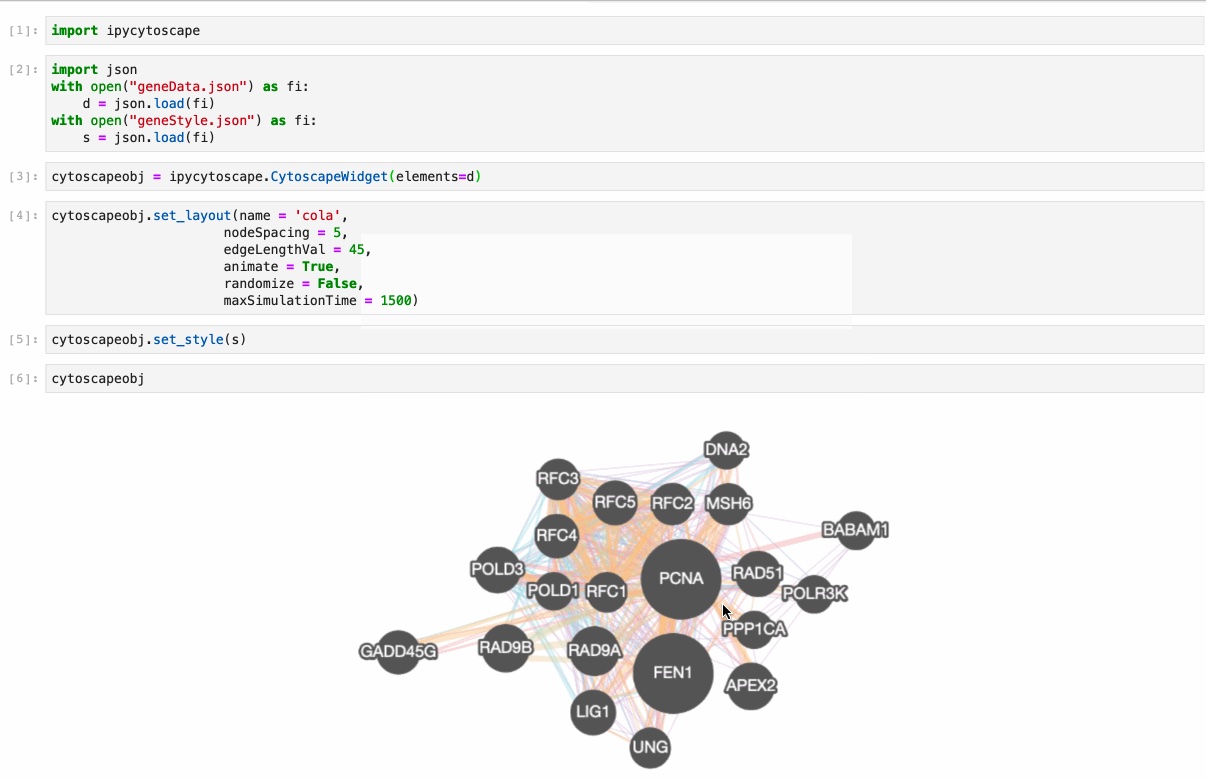

Supplement: btad031_Supplementary_Data [file btad031_supplementary_data.zip › Cytoscape 2023 update - supplementary matterials/Fig S1.jpg]

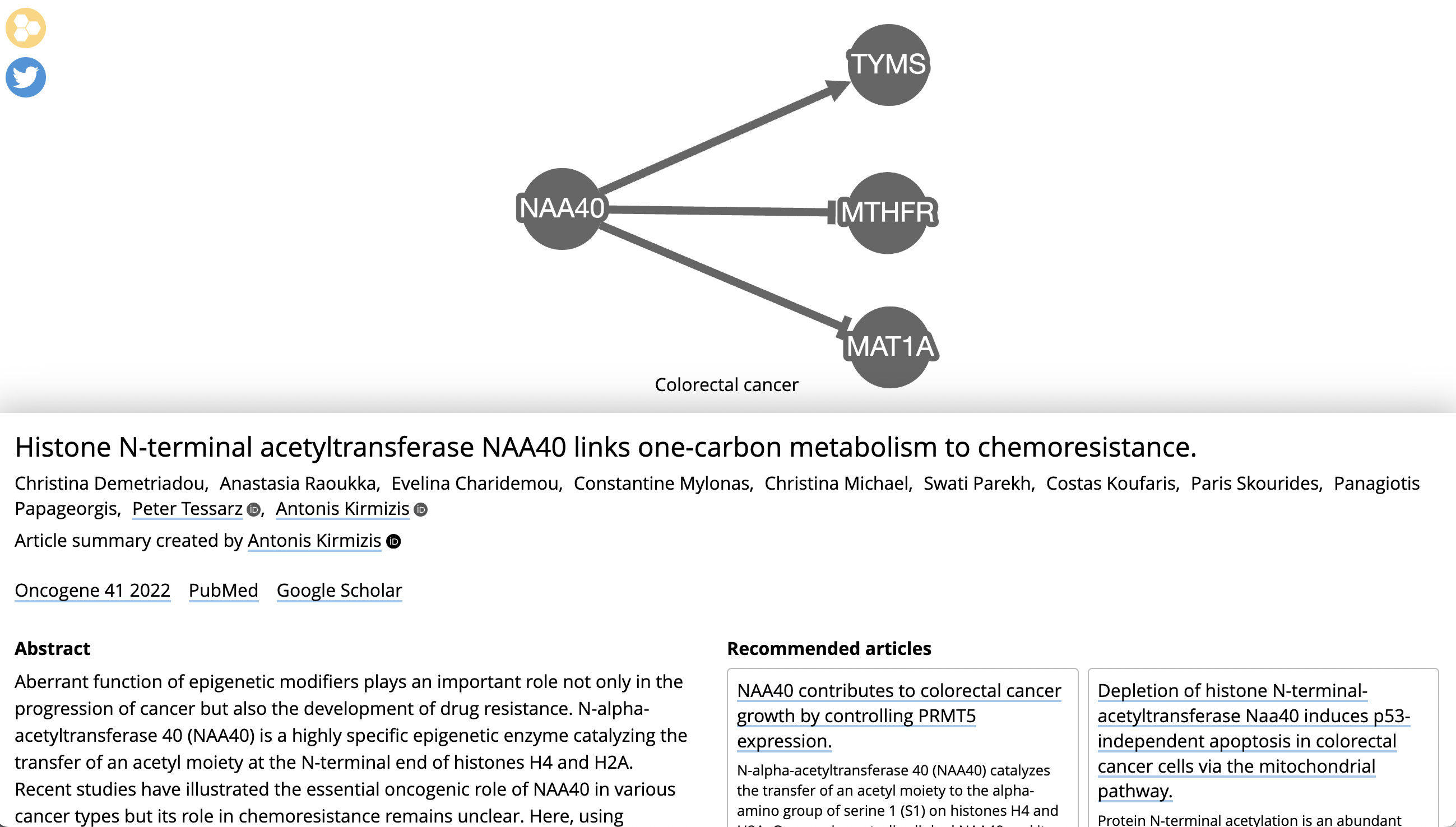

Supplement: btad031_Supplementary_Data [file btad031_supplementary_data.zip › Cytoscape 2023 update - supplementary matterials/Fig S2.png]

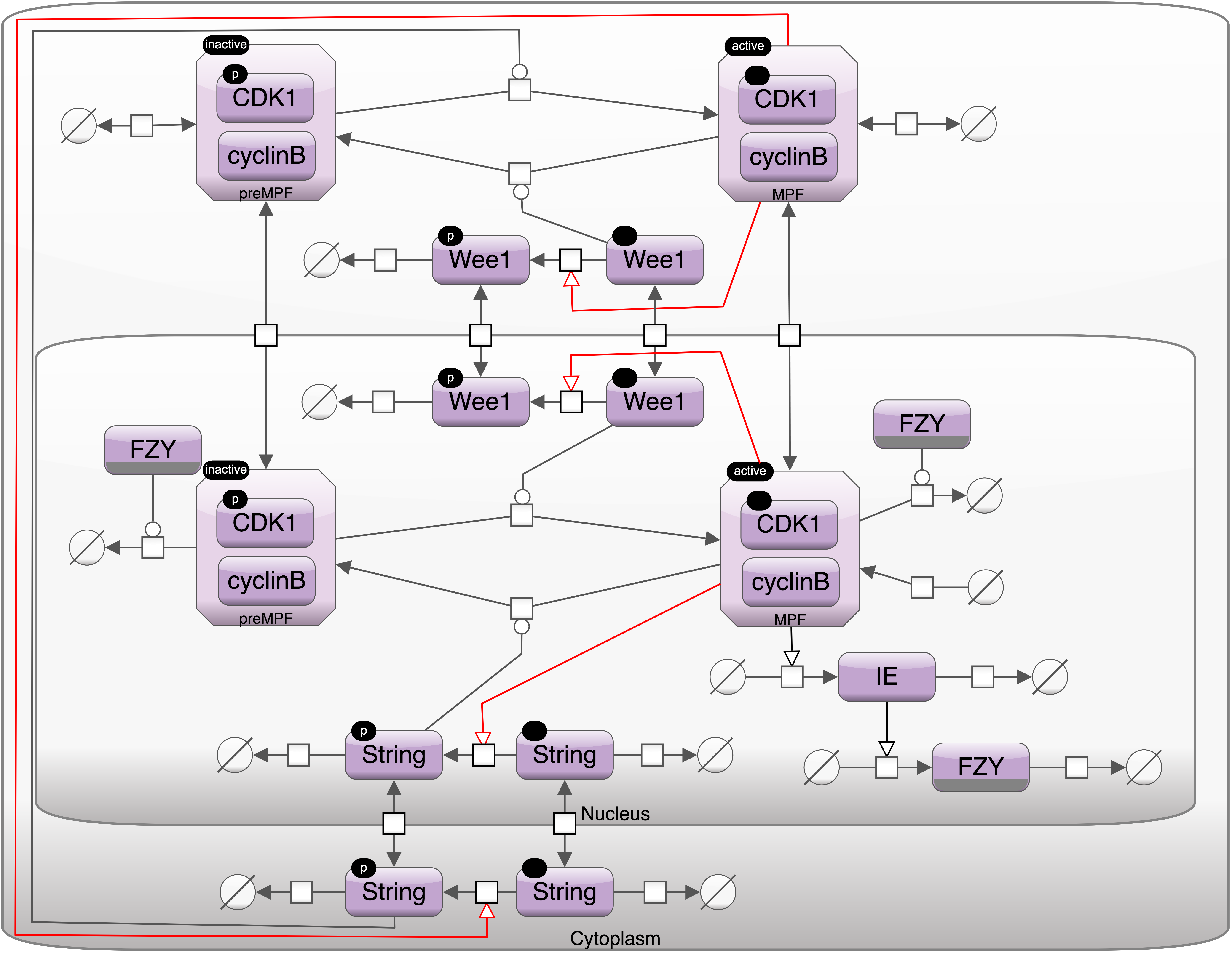

Supplement: btad031_Supplementary_Data [file btad031_supplementary_data.zip › Cytoscape 2023 update - supplementary matterials/Fig S3.png]
